# Evolution of Methylsilsesquioxane: From Hydrolytic Polycondensation Product to Xerogel

**DOI:** 10.3390/polym17030279

**Published:** 2025-01-22

**Authors:** Ivan B. Meshkov, Nadezhda G. Mazhorova, Artem V. Bakirov, Sergey G. Vasil’ev, Aleksandra A. Kalinina, Aleksandra V. Bystrova, Aziz M. Muzafarov

**Affiliations:** 1 A.N. Nesmeyanov Institute of Organoelement Compounds, Russian Academy of Sciences, 119334 Moscow, Russia; 2 Enikolopov Institute of Synthetic Polymeric Materials, Russian Academy of Sciences, 117393 Moscow, Russia; 3 Federal Research Center of Problems of Chemical Physics and Medicinal Chemistry, Russian Academy of Sciences, Chernogolovka, 142432 Moscow, Russia

**Keywords:** polymethylsilsesquioxane, hydrolytic polycondensation, sol, hydrogel, xerogel

## Abstract

Silica fillers have been a cornerstone in chemical technology due to their versatility, availability, and ease of integration into various formulations. Recent advancements, including chlorine-free synthesis of alkoxysilanes, have paved the way for alternative materials like polymethylsilsesquioxane (PMSSO). This study explores the structural evolution and properties of a hydrophobic PMSSO xerogel, synthesized through hydrolytic polycondensation of methyltriethoxysilane (MTEOS). PMSSO exhibits exceptional hydrophobicity, high specific surface area, and compatibility with polymer matrices, making it a promising filler for applications in rubber products, lubricants, and cosmetics. We developed a straightforward synthesis method for producing PMSSO xerogel that avoids toxic solvents and organochlorosilanes, ensuring safety and sustainability. The reaction conditions, particularly the amount of alkali and neutralization parameters, were found to significantly influence the properties of the final xerogels, such as specific surface area. Optimization of the synthesis parameters allow for obtaining PMSSO xerogels with a specific surface area about 600 m^2^/g. These findings underscore PMSSO’s potential as a versatile, eco-friendly alternative to conventional silica fillers, offering tailored properties for diverse industrial applications.

## 1. Introduction

Silica fillers have long been a cornerstone in various sectors of modern chemical technology, owing to their versatility and established production practices. Notable advancements, such as the development of “green tires” [1], highlight the ecological and functional benefits of replacing traditional carbon black fillers with precipitated silica. Beyond tire technology, hydrophobic aerosil (fumed silica) has gained widespread use as a viscosity regulator in industries ranging from paints, varnishes, and lubricants to cosmetics and pharmaceuticals [2]. This dominance is primarily attributed to silica fillers’ availability, favorable properties, and ease of integration into diverse formulations. The study of various methods for introducing silica fillers [3,4,5,6,7] has remained relevant for the past 50 years, as the efficiency of silica fillers largely depends on the method of their incorporation, which determines the uniformity of particle distribution, interfacial interaction with the polymer matrix, and, consequently, the mechanical, thermal, and barrier properties of the final material.

The approaches currently being developed for direct chlorine-free synthesis of alkoxysilanes [8,9,10,11] create a favorable environment for the emergence of alternative materials, such as polymethylsilsesquioxane (PMSSO), that offer competitive advantages while addressing limitations of traditional fillers. PMSSO, with its general formula [CH3SiO_1.5_]_∞_, is synthesized via hydrolytic condensation of methyltrialkoxysilane. This process yields a fine, white, highly porous powder with particle sizes ranging from 1 to 100 microns and a bulk density of 40–250 g/L. Its exceptional properties, including hydrophobicity, sufficiently high specific surface area, and compatibility with polymer matrices, make PMSSO a promising candidate for applications in rubber products, lubricants, and cosmetic formulations.

In prior studies [12], PMSSO demonstrated strong potential as a hydrophobic filler in rubber compounds. Comparative analyses revealed that vulcanizates containing PMSSO exhibited mechanical properties comparable to those with carbon black, while significantly surpassing them in thermal stability. Additionally, PMSSO’s intrinsic hydrophobicity eliminates the need for surface modifications in rubber applications, offering potential cost savings in production. The material’s chemical inertness, fire resistance, and non-toxicity further enhance its appeal for industrial and consumer applications. These attributes, combined with the ability to tailor PMSSO properties through synthesis parameters or comonomer inclusion, position it as a versatile and sustainable alternative to conventional silica fillers.

The objective of this study was to explore the evolution of the structural characteristics and properties of PMSSO from hydrolytic polycondensation of methyltriethoxysilane (MTEOS) to solid hydrophobic xerogels and determine the influence of all stages of the process on the properties of the final materials.

## 2. Materials and Methods

PMSSO sol: A 314.8 g (1.77 mol) quantity of MTEOS was added, while stirring, to a solution of 70.7 g (1.77 mol) of sodium hydroxide in 600 mL of water. The mixture was stirred for approximately 20 min until homogenization. As a result, 984 g (99.8% of the theoretical yield) of PMSSO sol solution with a 17% solid content was obtained.

PMSSO hydrogel: An 8.1 g (0.135 mol) quantity of acetic acid in the form of a 10% solution in 73 mL of water was added, while stirring, to 100 g of the PMSSO sol solution. The mixture was left to allow the gel to mature for 24 h. Afterward, hydrogel was washed on a Schott filter until the wash water showed a neutral reaction to phenolphthalein. As a result, 80 g of PMSSO hydrogel with a solid content of 6.5% was obtained.

Drying of the PMSSO hydrogel was carried out in a laboratory drying oven (BINDER GmbH, Tuttlingen, Germany) at a temperature of 150 °C for 8 h to a constant weight or using a laboratory spray dryer (Büchi Labortechnik AG, Flawil, Switzerland) from a 2% suspension in acetone at a temperature of 70 °C.

The true density ($\rho_{t}$, g/cm^3^) of PMSSO xerogels at 20 °C was determined using a small chamber with a volume of 4 cm^3^ on a Pycnomatic ATC gas pycnometer (Thermo Fisher Scientific Inc., Waltham, MA, USA). The true density for xerogel samples obtained from MTES and dried by different methods ranged from 1.34 to 1.37 g/cm^3^.

The bulk density of PMSSO xerogels ($\rho_{b}$, g/cm^3^) was calculated using the formula

(1) $$\rho_{b}=\frac{m_{x}}{V_{x}}$$

where $m_{x}$ is the mass of the PMSSO xerogel sample with a volume of $V_{x}$.

The porosity (ε, %) of PMSSO xerogels was determined using the formula

(2) $$\varepsilon =\left(1-\frac{\rho_{b}}{\rho_{t}}\right)\times 100$$

The thermal stability of PMSSO xerogels in air was studied using a Paulik–Paulik–Erdey derivatograph system (MOM, Budapest, Hungary) at a heating rate of 5 °C/min within the temperature range of 20–700 °C.

The specific surface area (S, m^2^/g) of PMSSO xerogels was determined using the BET method with a Sorbi-MS analyzer (Meta, Novosibirsk, Russia).

The surface structure of PMSSO powders was examined by scanning electron microscopy (SEM) using a JCM-6000 electron microscope from JEOL (Tokyo, Japan). Particle sizes were estimated randomly based on the most representative cross-sections and projection diameters. The relative error in determining linear dimensions was 10–20%.

The compositions and structures of the PMSSO sol and hydrogel were analyzed using solid-state NMR spectroscopy. The ^29^Si NMR spectra were registered on a Bruker Avance III 400 spectrometer. The analysis of the PMSSO sol was carried out in d-ethanol upon addition of Cr(acac)_3_. The values of the chemical shifts for particular units were as follows: MeSiOH(OEt)_2_ −41.6 ppm, (MeSiO_0.5_(OH)_2_) −49.9 ppm, (MeSiO_0.5_(OEt)_2_) −51.7 ppm, (MeSiO(OH)) ranging from −57.8 to −58.9 ppm, (MeSiO_1.5_) ranging from −64.0 to −67.5 ppm.

The analysis of the PMSSO gel was carried out using a solid-state probe under magic-angle spinning with the frequency of 8 kHz, cross-polarization, and 1 H decoupling. The value of a chemical shift for MeSiO_0.5_(OH)_2_ unit was −46.0 ppm, that for MeSiO(OH) −55.2 ppm, and that for MeSiO_1.5_ −66.3 ppm.

Using X-ray radiation at 8 keV (with a wavelength of 1.445 Å and resolution dE/E = 10^–3^), we conducted in situ small-angle X-ray scattering (SAXS) experiments at the BIOMUR station of Kurchatov synchrotron (National Research Center “Kurchatov Institute”). The photon flux was 10^9^ s^−1^, and the beam spot on samples was 0.5 × 0.3 mm^2^; diffraction patterns were recorded with a Dectris Pilatus 1M detector. The sample-to-detector distance was approximately 2500 mm, and a silver behenate standard was used. Exposure was 300 s. Data processing was performed using Fit2D software (v. 17.006). Further evaluation was carried out using ATSAS 2.8.4 software package. To calculate specific surface, we estimated true material density as 1.35 g/cm^3^ and porosity as 88%.

## 3. Results and Discussion

The synthesis of polymethylsilsesquioxane (PMSSO) xerogels involved the following stages: (1) alkaline hydrolysis of MTEOS and heterofunctional condensation of intermediates to form a sol; (2) partial neutralization of a sol with inorganic acid to produce a hydrogel; (3) washing of a hydrogel to remove low-molecular byproducts and residual alkali, resulting in a neutral hydrogel; (4) drying the hydrogel (oven or spray drying).

Virtually every stage of this process has a significant impact on the properties of the final product, that is why we carefully studied all steps of the PMSSO transformation.

**Figure 1:**
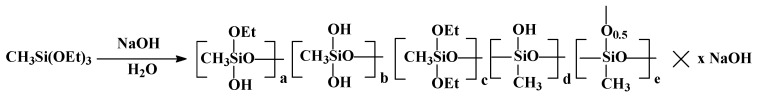
The synthesis scheme for PMSSO sols (x = 0.75 or 1—NaOH/MTEOS mole ratio).

According to this scheme, the first stage involves producing a soluble PMSSO sol through the hydrolysis of MTEOS in an alkaline medium. In this process, methyltriethoxysilane is treated with an aqueous alkali solution to obtain a homogeneous PMSSO sol solution (Figure 1).

As a result, stable sols with a dry residue of approximately 17% by weight are obtained, which remain stable over long storage periods. The actual structures of such sols can be evaluated using high resolution ^29^Si NMR spectroscopy. Previously [13] we determined the ratio of the structural units to be **a**:**b**:**c**:**d**:**e** = 0.3:1.2:0.5:3:1 for x = 0.75.

**Figure 2:**
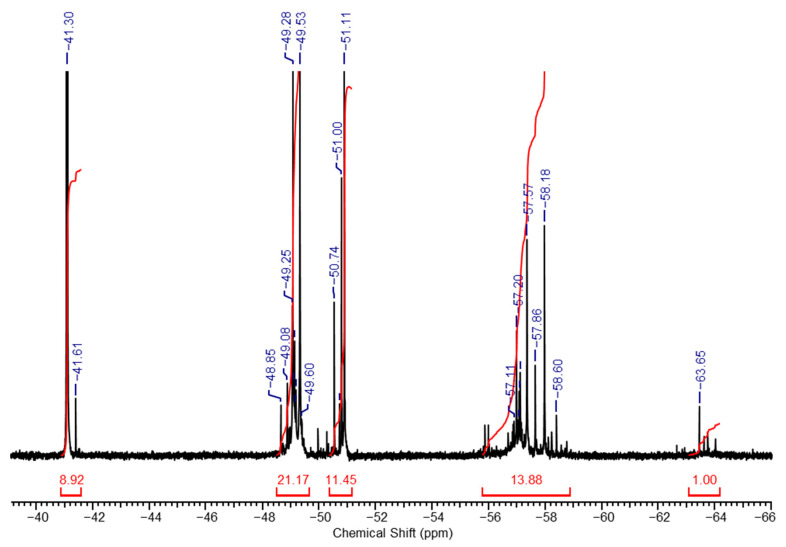
High-resolution *^29^*Si NMR spectrum of a in d-ethanol for x = 1.

The stability of the sol, as well as the ratio of structural units in its composition, depends on the alkali/MTEOS ratio used during hydrolysis. Upon the increase in alkali amount (x = 1), the sol composition remains identical, but the unit’s ratio changes significantly. A shift is observed towards an increase in lower molecular weight hydrolysis products, as confirmed by the ^29^Si NMR spectrum shown in Figure 2. It clearly demonstrates another structural units ratio, namely, **a**:**b**:**c**:**d**:**e** = 9:21:11:14:1. For instance, the amount of the low molecular weight partial hydrolysis product (unit **a**) increased approximately 30-fold.

When comparing the stability (time till visually detected gelation) of the sol, at x = 0.75, it remains stable for about a year, whereas at x = 1, the sol remains stable for a significantly longer period (over three years). The stability of the sols was evaluated during storage at room temperature (25 °C) in hermetically sealed containers.

**Figure 3:**
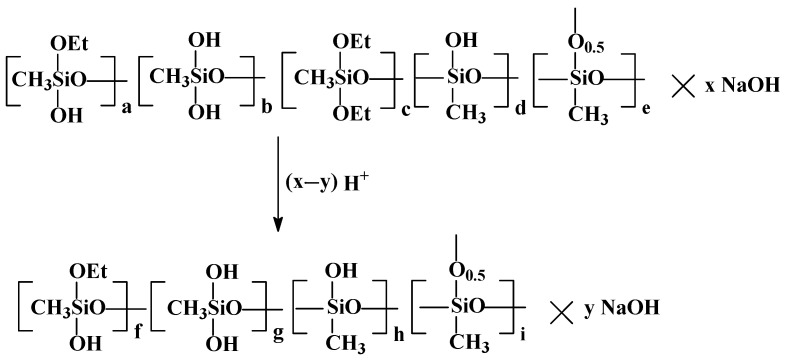
The scheme of partial neutralization of PMSSO sols to obtain hydrogels (x = 1; y = 0.2–0.3).

The next stage in the preparation of the xerogel is the partial neutralization of PMSSO sols with any inorganic acids, such as hydrochloric, sulfuric, acetic, etc., which were added as a 10% aqueous solution, leading to the formation of PMSSO gel. The scheme is shown in Figure 3.

It was determined that to achieve xerogels with the maximum surface area (around 600 m^2^/g), acid should be added to the PMSSO sol in a ratio of 0.7–0.8 per 1 NaOH unit of sol (as shown by the coefficient (x−y) in Figure 3). Under these conditions, gel formation occurs rapidly, within 5 min, resulting in an optimal xerogel with high surface area and fine gel particle size. However, if the sol is fully neutralized, the surface area decreases significantly (to about 30 m^2^/g), and the gel becomes denser.

**Figure 4:**
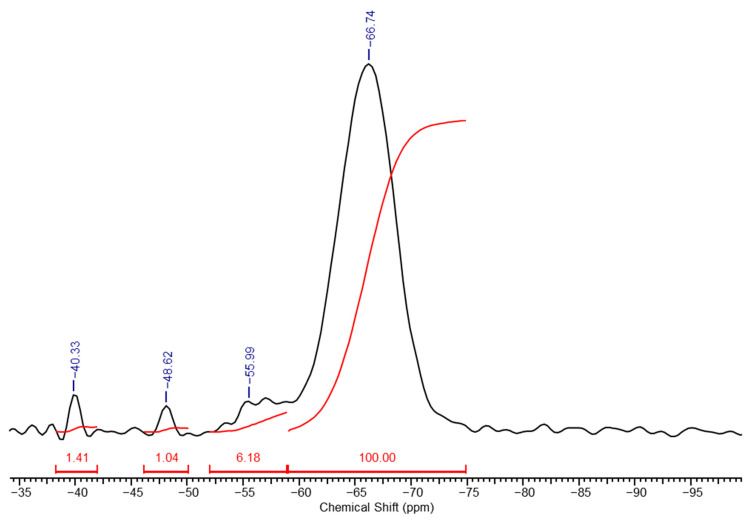
High-resolution solid-phase ^29^Si NMR spectrum of a PMSSO hydrogel (x = 1, y = 0.3).

In the high-resolution solid-phase ^29^Si NMR spectrum of a PMSSO hydrogel (Figure 4), distinct differences are observed compared to the spectrum of its precursor (Figure 2). The signal from the methylsilsesquioxane part (unit **e**) increases sharply, while the signals from the other units decrease significantly and diethoxysilane units disappear completely due to processes of acidic hydrolysis of the ethoxysilane groups with the release of ethanol and the formation of hydroxyl groups, which in turn interact with another ethoxy- or hydroxyl groups with the formation of siloxane bond and release of ethanol or water, respectively. Based on the spectrum, the ratio of units **f**:**g**:**h**:**i** was determined to be 1.4:1:6.2:100. The maturation of the final gel occurs within several hours after the gel formation begins (depending on the amount of acid used). For example, for x = 1, y = 0.3 maturation took approximately 2–3 h.

**Figure 5:**
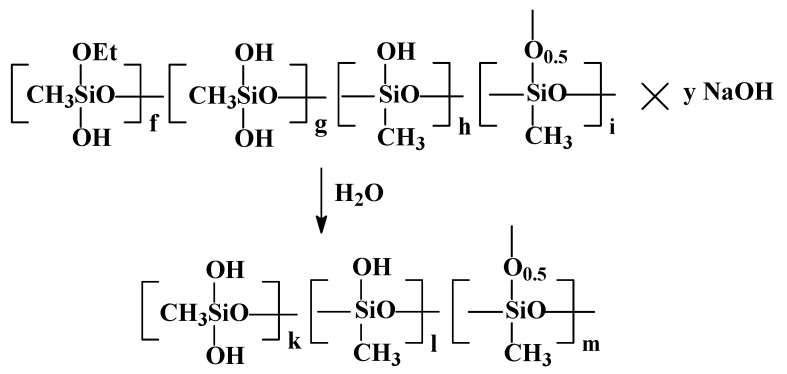
The schematic of washing of PMSSO hydrogels to remove low-molecular impurities and residual alkali.

The third stage in the method for preparing PMSSO xerogel is the washing of the gel, obtained in the second stage, to remove the residual alkali. The schematic representation of this process is shown in Figure 5.

**Figure 6:**
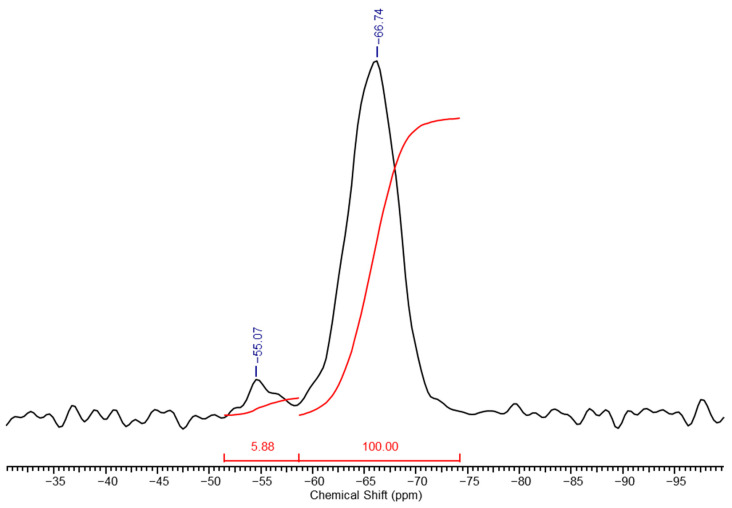
High-resolution solid-phase ^29^Si NMR spectrum of the washed PMSSO hydrogel.

The washing is carried out until the wash water shows a neutral reaction with phenolphthalein. The resulting gel was also studied using ^29^Si NMR spectroscopy, and its spectrum is presented in Figure 6. The spectrum clearly shows the disappearance of the signals from units **f** (due to full hydrolysis of ethoxy groups), with the resulting ratio of units **k** + **l**:**m** being 6:100.

**Figure 7:**
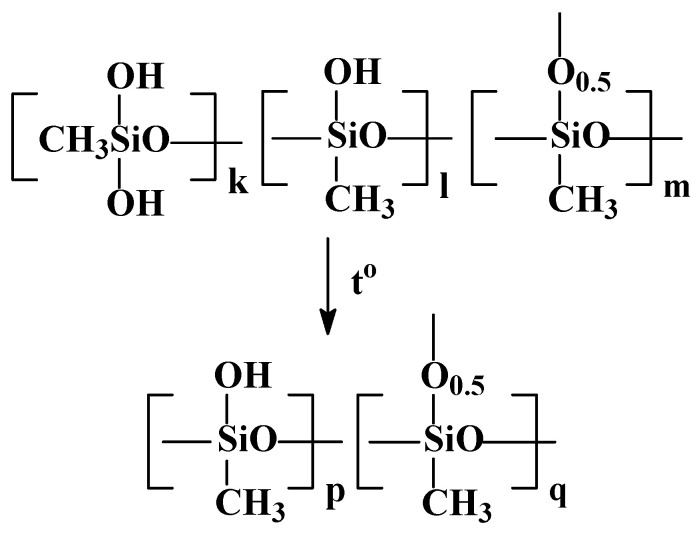
The schematic of drying of PMSSO hydrogels.

The final stage in the preparation of the xerogel is drying, which is schematically shown in Figure 7.

As mentioned in the general scheme, there are several drying methods available. The first and simplest is thermal drying in an oven at various temperatures (e.g., from 150 to 250 °C) for different durations (e.g., from 2 to 8 h).

**Figure 8:**
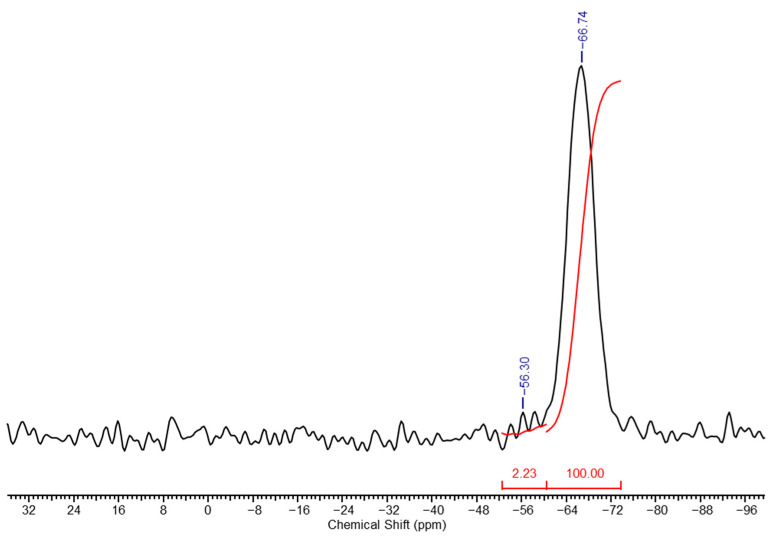
High-resolution solid-phase ^29^Si NMR spectrum of PMSSO xerogel (oven drying).

PMSSO xerogels dried using this method are powders with a relatively high surface area, around 600 m^2^/g. The high-resolution solid-phase ^29^Si NMR spectrum of xerogel (Figure 8), obtained by oven drying (and as will be shown later, by any method), shows the signals from the units **k** with two hydroxyl groups on the silicon atom completely disappeared, i.e., conversion almost approaches quantitative; however, due to the rigidity of the structure and spatial hindrance, some hydroxyl groups remain (units **p**).

The ratio of units **p** and **q** determined from the spectrum was 2.2:100.

It was also found that the surface area depends on the quality of the initial gel, specifically on the acid ratio during gel formation, shaping, and maturation. In other words, for denser gels dried using this method, the surface area was quite small (around 30 m^2^/g).

**Figure 9:**
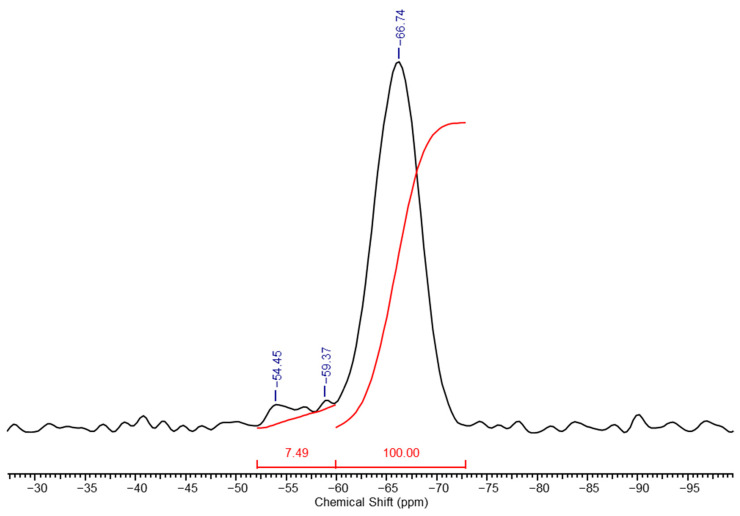
High-resolution solid-phase ^29^Si NMR spectrum of the PMSSO xerogel (spray drying from acetone).

The next method is laboratory spray drying, performed using a Buchi brand laboratory dryer. A series of experiments were conducted to study the influence of the solvent on the properties of the xerogel, and it was found that the choice of solvent did not affect the surface area, which in all cases did not exceed 600 m^2^/g, matching the surface area of xerogels dried by the first method. However, the xerogels dried using laboratory spray drying showed differences in the ratio of units compared to those dried thermally, due to the milder conditions. The number of units **p** was four times higher, resulting in a **p**:**q** ratio of 7.5:100, as demonstrated by the comparison of ^29^Si NMR spectra of the corresponding xerogels. Figure 9 shows the spectrum of the xerogel dried by the second method.

**Figure 10:**
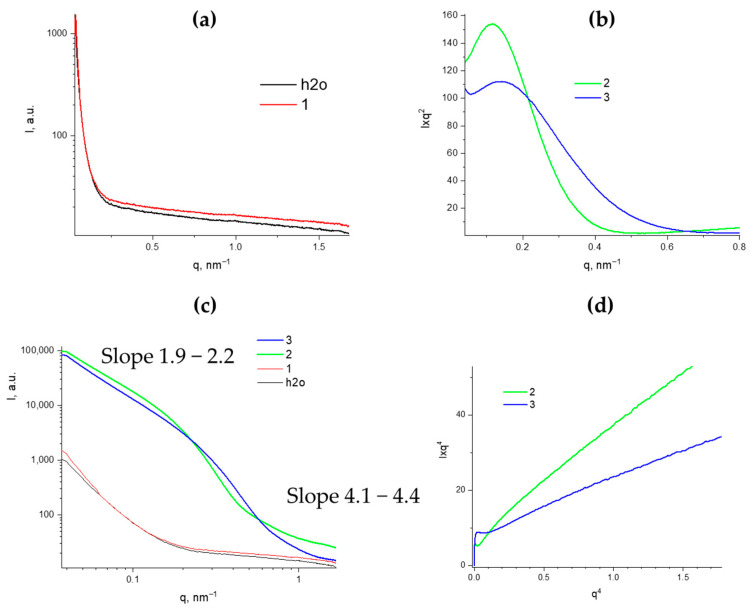
(**a**)—SAXS curves for the initial sol (1) and aqueous buffer. (**b**)—SAXS curves in Kratky coordinates for gels before (2) and after (3) washing off sodium acetate. (**c**)—SAXS curves in double logarithmic coordinates. (**d**)—Porod plots for gels before and after washing off sodium acetate.

The structural transformations of the PMMSO were also studied using X-ray structural analysis. The initial PMSSO sol was analyzed in transmission mode. The resulting curve, after subtracting the buffer, shows values close to zero across the entire low-angle scattering vector range (Figure 10a), indicating that this solution is true and no aggregates form, despite its high concentration (17 wt.%).

Further studies were conducted on PMSSO hydrogels before and after washing off residual sodium acetate. The resulting curves were plotted in Kratky coordinates to determine the invariant, in double logarithmic coordinates to evaluate the fractal dimension of aggregates in the sample, and in Porod coordinates to assess the specific internal surface area (Figure 10b–d).

In the double logarithmic plots, the presence of two structural types is clearly visible: in the small **q** range (0.04–0.15 nm^−1^) and in the medium **q** range (0.15–0.7 nm^−1^), which differ significantly in fractal dimension. Aggregates with sizes of 50–150 nm exhibit a fractal dimension of approximately 2, indicating a disk-like shape [14], with a radius of gyration of 30–35 nm. For particles up to 40 nm in size, the slope of the linear approximation exceeds 4, characterizing these particles as compact spheres with rough surfaces.

The obtained SAXS curves were plotted in coordinates I × q^4^ from q^4^ to determine the Porod constant k to calculate the specific surface area value using the following Porod equation:

$$\frac{S}{m}=\frac{\pi }{\rho }\cdot \frac{k}{Q}\cdot \phi_{p}(1-\phi_{p}),$$

where S/m is the specific volume in m^2^/g, *ρ* is the macroscopic density of the sample, k is the Porod constant, defined as the point of intersection of the ordinate axis with the linear approximation of the curve plotted in coordinates I × q^4^ from q^4^, ϕ_p_ is the porosity (pore volume fraction in the sample), and Q is the invariant calculated from the small-angle Kratky plot I × q^2^ of q using the following equation:

$$Q=\int_{0}^{\infty }I(q)\cdot q^{2}\cdot dq$$

While the invariant values for hydrogels before and after washing are similar, the Porod constant values differ significantly, resulting in specific surface areas of 380 m^2^/g and 660 m^2^/g, respectively.

Fractal dimensions between 1.7 and 2.1 are typical for clusters formed by kinetic growth processes, i.e., irreversible aggregation far from equilibrium [15]. For silicon and organosilicon compounds, the critical particle size for transitioning into the kinetic aggregation regime is approximately 12 nm.

### Effect of Drying Conditions on Structure

**Figure 11:**
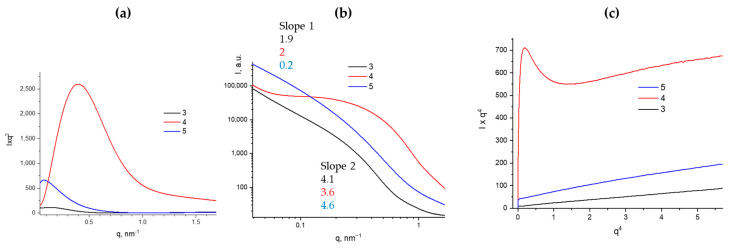
(**a**)—SAXS curves in Kratky coordinates for gels dried under different conditions (3—washed hydrogel; 4—oven dried xerogel; 5—spray dried xerogel); (**b**)—SAXS curves in double logarithmic coordinates with characteristic slopes indicated; (**c**)—Porod plots.

The influence of drying conditions on the structure was studied by comparing small-angle X-ray scattering (SAXS) data for samples of washed gel dried in a drying oven and using laboratory spray drying from an acetone suspension. As shown in Figure 11, the drying method does not significantly affect the structure of the particles themselves, with the fractal dimension in the higher-angle range remaining close to 4. However, the shape of the aggregates changes significantly. Spray drying from acetone suspension allows for the preservation of a flattened (disk-like) shape of the aggregates during drying, whereas oven drying without transferring to a water suspension enhances the collapsing effect. This is manifested by a decrease in the fractal dimension to below 1, indicating the attainment of the Guinier limit. Simultaneously, the invariant value increases significantly, indicating an increase in the roughness of the sample, and the specific internal surface area reaches a record value of 990 m^2^/g.

In contrast, the powder obtained by spray drying of the hydrogel suspension in acetone does not show high invariant values or specific surface areas—reaching only 550 m^2^/g, see Table 1. This significant difference can be explained by the presence of a large number of closed pores in oven dried xerogel sample, likely due to cyclization processes occurring during gel drying without the use of a spray dryer. Closed pores within the studied size range also contribute to the specific surface area derived from SAXS data; however, this additional contribution cannot be accessed by the BET adsorption method, which explains the substantial difference observed. This effect has been previously described elsewhere [16,17].

The thermogravimetric analysis data of PMSSO xerogels indicate their high thermal stability. The thermal resistance of the xerogels was found to be 400 °C.

## 4. Conclusions

We demonstrated a method for obtaining a hydrophobic organosilicon xerogel that is relatively simple to implement, involves a small number of technological steps, and avoids the use of problematic substances such as flammable or toxic solvents and organochlorosilanes.

Optimal conditions were identified for producing xerogels with high surface area and fine gel particle size. It has been shown that depending on the reaction conditions, xerogels with different surfaces and morphologies can be obtained. The properties are most significantly influenced by the amount of alkali used in the hydrolytic polycondensation reaction, as well as the neutralization conditions, while the influence of drying conditions is less significant.

## Figures and Tables

**Table 1:** Material characteristics, measured at different stages of PMSSO xerogel preparation.

	BET	Bulk Density	Particle Size	SAXS
PMSSO Sample	S_sp_, m^2^/g	ρ, g/cm^3^	d, μm	S_sp_, m^2^/g
Hydrogel (2)	-	-	-	380
Washed hydrogel (3)	-	-	-	660
Xerogel (oven dried) (4)	620	0.163	4–120	990
Xerogel (spray dried) (5)	580	0.163	4–120	550

## Data Availability

The original data reported in this study are available from the corresponding author on reasonable request.
